# Dual diagnosis of autosomal dominant polycystic kidney disease and sickle cell disease in a teenage male

**DOI:** 10.1007/s00467-023-05873-6

**Published:** 2023-01-16

**Authors:** Quinn Stein, Kathleen Herman, Jennifer Deyo, Colleen McDonough, Michelle S. Bloom, Asifhusen Mansuri

**Affiliations:** 1 Natera, Inc., Austin, TX USA; 2 Augusta University Medical Center, Augusta, GA USA

**Keywords:** Sickle cell disease, Polycystic kidney disease, Genetic testing, Kidney cysts

## Abstract

**Background:**

Sickle cell disease (SCD) and autosomal dominant polycystic kidney disease (ADPKD) are relatively common genetic conditions with considerable overlap in clinical presentation. In addition to similarities between the signs and symptoms in sickle cell nephropathy and ADPKD, more than half of SCD patients have kidney cysts. The co-occurrence of these two diseases has not been previously reported in the literature.

**Case diagnosis/treatment:**

A 16-year-old Black male with SCD had bilateral kidney enlargement and multiple simple cysts on ultrasound. Although kidney cysts are significantly more common in individuals affected with SCD, genetic testing with a broad kidney gene panel was performed to explore the possible presence of another underlying genetic cause of his cysts, in addition to SCD. A dual diagnosis of SCD and ADPKD was made following the identification of two copies of the common pathogenic sickle cell *HBB* variant (c.20A > T, p.Glu7Val) and a pathogenic missense variant in *PKD1* (c.8311G > A, p.Glu2771Lys).

**Conclusions:**

SCD and ADPKD differ in pathophysiological mechanisms and treatment regimens. As such, it will be paramount for this teenager to be closely monitored for signs of diminished kidney function and to be co-managed as he transitions to adult care to ensure proper treatment and management. Early identification of individuals with both SCD and a co-occurring condition is crucial to ensuring proper clinical management. Furthermore, identifying and reporting additional patients with SCD and ADPKD dual diagnoses will help us to understand the co-occurring disease course and optimal treatments.

## Background

Sickle cell disease (SCD) and autosomal dominant polycystic kidney disease (ADPKD) are common genetic conditions with partially overlapping kidney phenotypes (Table 1). SCD is caused by a specific pathogenic variant in the *HBB* gene while ADPKD is most commonly caused by variants in *PKD1* or *PKD2*. Despite the relatively high prevalence of these conditions (ADPKD affects approximately 1 in 1000 people; SCD occurs in approximately 1 out of every 365 Black individuals [1, 2]), the co-occurrence of these two conditions does not appear to have been previously reported.

**Table 1:** Comparison of common kidney manifestations and select extrarenal findings in sickle cell disease and autosomal dominant polycystic kidney disease [1, 5, 11, 12, 19, 20]

Disease symptoms	Sickle cell disease	ADPKD
Kidney cysts	x	x
Progressive chronic kidney disease	x	x
Hematuria	x	x
Hypertension	x	x
Kidney Ischemia	x	x
Kidney and/or glomerular enlargement	x	x
Kidney, flank, or back pain	x	x
Nocturia or enuresis	x	x
Reduced urinary concentrating abilities	x	x
Urinary tract infection	x	x
Renal cell carcinoma or carcinoma complications	x	x
Intracranial aneurysms	x	x
Susceptibility to acute kidney injury	x	x
Tubulointerstitial disease	x	
Glomerulopathy	x	
Impaired kidney potassium excretion	x	
Renal papillary necrosis	x	
Nephrolithiasis		x

A handful of studies have reported the co-occurrence of ADPKD and sickle cell trait (SCT), the presence of a single pathogenic variant in the *HBB* gene, along with variants in either *PKD1* or *PKD2*. The first report, in 1994, noted that the onset of kidney failure occurred approximately 10 years earlier in Black people with both ADPKD and SCT, compared to ADPKD alone [3]. In 2011, Peces et al. described a proband with frequent hematuria, increased kidney volume, and early-onset kidney failure requiring dialysis at age 39. That study hypothesized that repeated episodes of localized sickling and venous occlusion could lead to chronic microvascular damage, which could cause chronic hypoxia and tubulointerstitial fibrosis [4]. As such, it is possible that SCT (and potentially SCD) coexisting with other conditions that affect kidney microvasculature (e.g., ADPKD) could act confluently to accelerate kidney damage.

Kidney cysts are significantly more common in individuals affected with SCD than in the general population; approximately 58% of individuals with SCD are reported to have kidney cysts with 28% having bilateral cysts [5]. The commonality of cysts in individuals with SCD can make it difficult to determine if the presence of cysts is due to SCD alone, or in part, due to an additional cystic disease like ADPKD, particularly in its early stages.

## Case diagnosis/treatment

A 16-year-old Black male with sickle cell disease (SCD) initially presented to the pediatric nephrology service nine years prior with nocturia. Ultrasound evaluations at age 7 revealed three simple cysts on each kidney (largest cyst right kidney 0.8 × 0.6 × 0.9 mm; largest cyst left kidney 1.4 × 1.5 × 1.2 cm). The patient’s right and left kidney length measured 10 cm and 10.1 cm (98–99th percentile per patient height), respectively. Kidney function was within normal limits (BUN 13 mg/dL, SCr 0.3 mg/dL). The patient was maintained on desmopressin for enuresis for several years. Monitoring of the bilateral cysts via retroperitoneal ultrasounds over subsequent visits revealed interval increase in size and number. Sickle cell nephropathy with proteinuria was managed by initiating ACE inhibitor treatment. For management of SCD, the patient had been treated with amoxicillin until age 5, monthly blood transfusions for primary stroke prevention, and a concomitant iron chelator to prevent iron-related toxicities. As an adolescent, the patient was transitioned to exchange transfusions and hydroxyurea (HU) therapy and monitored for internal carotid artery stenosis that can arise as a part of the natural history of the disease with Doppler ultrasound and MRI/MRA.

Due to an increasing number of kidney cysts over time, believed to be beyond what would be expected in SCD [5], genetic testing with a broad 385 kidney gene panel (the Renasight™ Test, Natera Inc., Austin, TX) was performed to identify underlying genetic causes, in addition to SCD, which may be related to the kidney cysts. Two copies of the common pathogenic sickle cell *HBB* variant (NM_000518.5:c.20A > T; p.Glu7Val) were identified, as well as a pathogenic missense variant in *PKD1* (NM_001009944.3:c.8311G > A; p.Glu2771Lys) [6, 7]. These genetic findings confirmed a dual diagnosis of ADPKD and SCD in this young man.

This patient is the first member of the family to have genetic testing performed; however, both of the patient’s parents are obligate carriers for SCD. After genetic testing in the proband, the patient’s father reported a diagnosis of kidney disease and has an unknown family history. Neither parent has been evaluated for the *PKD1* variant; thus, it is unknown if the variant identified in the proband is inherited or *de novo*, which occurs in 10–20% of ADPKD cases [1].

At age 16, the patient is an active teenager, attending high school. He requires an Individualized Education Plan for reading, comprehension, and test-taking. He regularly attends classes except for monthly transfusions. He is generally healthy; however, in the past year, he presented to the Emergency Department twice with sickle cell crises. The family has been extremely compliant and supportive of the patient’s medical care.

Most recent imaging shows kidney lengths of 13.7 cm and 14.5 cm (right and left, respectively, both > 99th percentile) with numerous kidney cysts, the largest measuring 4.0 × 3.0 × 4.5 cm. Kidney function has remained stable over time with a glomerular filtration rate (GFR) of 141 mL/min/1.73 m^2^ (BUN 8 mg/dL, SCr 0.66 mg/dL) and no symptoms of hematuria. His blood pressure has been consistently measured within the normal range. Additional screening by MRA shows evidence of cerebral vasculopathy with mild stenosis of his right carotid artery. No acute or chronic changes to the cerebral cortex have been noted. There is no evidence of hepatic cysts by abdominal MRI at this time.

## Conclusions/discussion

Based on estimates of the prevalence of SCD and ADPKD alone, we estimate that the co-occurrence of SCD and ADPKD occurs in approximately 1:365,000 Black individuals [1, 2]. Despite the predicted relative commonality of the co-occurrence of these diseases, review of the literature did not yield any published reports of such cases. The paucity of literature reporting dual diagnoses of SCD and ADPKD could be due to (1) the attribution of cysts and kidney complications to SCD, negating exploration of additional causes of kidney cysts after an SCD diagnosis is made; (2) the shortened life expectancy historically associated with SCD, which could limit the full progression of PKD, and subsequent kidney failure; or (3) limited access to individuals with these conditions due to disparities in access to quality care for people with SCD, particularly in low-income and rural communities with limited resources [8].

ADPKD and SCD are distinct genetic conditions that affect kidney function, with differing underlying pathophysiologies. ADPKD, which is often diagnosed in adulthood, arises from defects in the mechanosensory and chemosensory roles of cilia in tubular cell growth and differentiation. Fluid-filled cysts arise as a result of abnormal cell growth from the dysregulation of these signaling pathways. Continued growth of these cysts can cause kidney damage, fibrosis and ischemia, and hematuria resulting from cyst hemorrhage [9, 10].

SCD is a hemoglobin disorder caused by the presence of hemoglobin S (HbS) which is poorly soluble when deoxygenated and polymerizes within red blood cells (RBCs). When fully deoxygenated, RBCs become sickled in shape, causing membrane damage. Kidney complications emanate from veno-occlusive events in the vasa recta causing impairment in medullary blood flow [11]. Prostaglandin-mediated hyperfiltration and iatrogenic interstitial nephritis further accelerate the evolution of chronic kidney disease. The independent effects of these conditions on the kidneys suggest that co-occurrence could intensify disease severity. As such, it will be important to closely monitor this patient for signs of diminished kidney function to enable appropriate management.

SCD is routinely diagnosed through newborn screening programs that enable early education and prophylactic management prior to the onset of symptoms. Prophylactic antibiotic treatment for pneumococcal infection in SCD patients could also prevent or reduce the frequency of urinary tract infections seen in ADPKD, which can worsen kidney function. However, like many individuals with SCD, the patient detailed in this report has not experienced infectious complications warranting life-long antibiotic therapy [12]. Individuals with SCD are also recommended to avoid dehydration and extreme temperature changes to decrease episodes of veno-occlusive crisis. HU treatment, which inhibits HbS polymerization, and thereby the sickling and adhesiveness of RBCs, is the mainstay of medication therapy for SCD that is associated with significant morbidity and mortality benefits [13]. Together, the immense progress in treating and preventing the complications of SCD has significantly extended the life expectancy of affected individuals.

Identification of this patient’s ADPKD diagnosis can enable management changes that might not have otherwise been considered. For instance, tolvaptan therapy is effective in slowing kidney function decline in adults at risk of rapidly progressing ADPKD [14]. Due to the early diagnosis of ADPKD in this patient, tolvaptan therapy has the potential to preserve kidney function long term. However, tolvaptan has not been extensively studied in individuals with SCD, and it could increase the risk of veno-occlusive crisis episodes as a result of dehydration through aquaresis. While tolvaptan is not yet approved for use in children, the knowledge of this patient’s ADPKD diagnosis at age 16 enables consideration of the treatment as he becomes eligible as an adult. Tools such as measurement of total kidney volume (TKV) may be useful in determining this patient’s risk of disease progression, and as such, whether tolvaptan may be appropriate [15, 16].

Diagnosis of ADPKD in this patient can also enable surveillance for extrarenal complications. For example, intracranial aneurysms (ICA) occur in 8% of patients with SCD and 9–12% of patients with ADPKD; therefore, regular screening is warranted for patients with concomitant risk factors [1, 17, 18]. Likewise, liver involvement is frequently seen in SCD, but screening for ADPKD should also consider polycystic liver disease which can cause compressive symptoms and pain. Altogether, close co-management of this patient between nephrology and hematology is warranted, particularly as he transfers from pediatric to adult care.

This case illustrates that the co-occurrence of SCD with other genetic conditions with overlapping symptoms can present complex management scenarios for adolescents as they transition into adulthood. While there is considerable overlap in kidney symptoms between SCD and ADPKD, the pathophysiology and treatment regimens for these conditions differ. The introduction of preventative medicine for SCD has reduced the incidence of early mortality yet increased the incidence of kidney failure as this population ages. As such, identification of co-occurring kidney conditions is imperative to ensuring proper management that can slow down disease progression. In particular, genetic testing can be an important tool in this process for patients with SCD who have ultrasonographic evidence of cysts. Reporting additional cases of SCD and ADPKD occurring as dual diagnoses will be important to increase our understanding of the disease course and optimal treatment strategies.

## Data Availability

Data availability statement: The authors confirm that the data supporting the findings of this study are available within the article.
